# Design of Tunable Terahertz Metamaterial Sensor with Single- and Dual-Resonance Characteristic

**DOI:** 10.3390/nano11092212

**Published:** 2021-08-27

**Authors:** Jiahao Yang, Yu-Sheng Lin

**Affiliations:** School of Electronics and Information Technology, Sun Yat-Sen University, Guangzhou 510006, China; yangjh59@mail2.sysu.edu.cn

**Keywords:** tunable metamaterial, split-ring resonators, THz sensor, high sensitivity

## Abstract

We present two types of refractive index sensors by using tunable terahertz (THz) metamaterial (TTM) based on two concentric split-ring resonators (SRRs) with different splits. By modifying the distance between SRRs and substrate, TTM shows tunable single- and dual-resonance characteristic. The maximum tuning range of resonance is 0.432 THz from 0.958 THz to 1.390 THz. To demonstrate a great flexibility of TTM in real application, TTM device is exposed on the surrounding ambient with different refractive index (*n*). The sensitivity of TTM can be enhanced by increasing SRR height, which is increased from 0.18 THz/RIU to 1.12 THz/RIU under the condition of *n* = 1.1. These results provide a strategy to improve the sensing performance of the metamaterial-based sensing device by properly arranging the geometric position of meta-atoms. The proposed TTM device can be used for tunable filters, frequency-selective detectors, and tunable high-efficiency sensors in the THz frequency range.

## 1. Introduction

Terahertz (THz) wave has many unique advantages compared with electromagnetic waves in other frequency bands. It has low photon energy and strong penetration ability similar to X-ray. Particularly, in biological detection, many biomolecules and organic molecules have unique responses in the THz frequency band, and the fingerprint spectrum of biological tissues can be collected by THz spectrum information. Thus, it can realize label-free, non-destructive, and non-contact detection [1,2]. Owing to it is non-toxic and non-destructive to human tissue, THz sensor provides a promising and real-time biological device for practical pharmacological applications [3]. These characteristics indicate that THz technology has great potential in the detection of biomolecules and chemical species. Owing to the unique characteristics of THz wave, optical devices based on THz metamaterial can be used to enhance the field–matter interaction, manipulating and controlling the incident THz wave, making this a hotspot topic in the physics, material science, and optoelectronics fields. 

Metamaterials are artificial composite materials with periodic or aperiodic arrangements to show particular electromagnetic characteristics that natural materials do not have, such as negative refractive index [4,5,6,7]. The equivalent electromagnetic parameters of metamaterial can be obtained by designing the metal, semiconductor, and dielectric structures in the subwavelength scale which make the interaction between the incident light to metamaterial [8,9]. The typical configuration of metamaterial are split-ring resonators (SRRs) [10], which was first proposed by Pendry et al. [11] in 1999 based on Maxwell’s equation. On the basis of Pendry et al., the metamaterial based on SRR was fabricated by Smith et al. [12], and they observed the negative refractive index phenomenon. Until now, many derivative structures have demonstrated—such as I-shaped SRR, U-shaped SRR, and 3D-SRR, and complementary structures [13,14,15,16,17,18]—which can be used to span the visible [19,20], infrared [21,22], and THz [23,24,25] spectra ranges. The electromagnetic characteristics of various metamaterials are Fano resonance [26], electromagnetically induced transparency effect [27], and spoof surface plasmons [28,29]. In view of the above merits of metamaterial, it can be used in widespread applications—such as absorbers [30], invisibility cloaks [20], filters [31], sensors [32], and so on. However, the resonant frequency of most of the above devices is immutable. In order to improve the flexibility of THz metamaterials, many tuning methods have been reported, including thermal annealing, laser pumping, electrical tuning, and micro-electro-mechanical systems (MEMS) technology [33,34,35,36,37,38,39,40,41,42]. Among these methods, MEMS technology can actively modify the resonant frequency by changing the geometric parameters of metamaterial unit cell [38,39,40,41,42]. It is not limited by materials and external environment, and has a wider tuning range of resonance [42]. 

In this study, we propose two designs of tunable THz metamaterial (TTM), which are composed of two concentric SRR (1-cut SRR) and two concentric electric SRR (3-cut SRR) configurations to denote as TTM-1 and TTM-2, respectively. Outer and inner SRRs have the same shape but different geometric parameters, which are named as SRR-1, SRR-2, SRR-3, SRR-4, respectively. By changing the height between SRRs and substrate, the electromagnetic responses of metamaterial are investigated. The height of a single SRR is changed to induce the resonance shift. To further explain the physical mechanism of the interaction in incident THz wave and metamaterial, the electric (E) and magnetic (H) fields distributions are analysed. Therefore, the height of SRR-2 and SRR-4 can be modified to realize two tunable resonances in different ranges, which make the TTM device with flexible tunability. Moreover, when the height between SRR-2 and SRR-4 to substrate is 4 μm, the sensitivity and figure of merit (FOM) of the TTM device are better compared with the height of 0 μm, which is suitable to be used as a refractive index sensor. In the next stage, when we start to fabricate the proposed device, it is important to pay attention to the difference between experiment and simulation. Surface roughness and tolerances in geometrical factors of designed patterns are key factors affecting the performance of device [43]. Such TTM designs provide an idea for tunable THz optoelectronics and can be further applied in the highly sensitive sensors.

## 2. Designs and Methods

**Figure 1:**
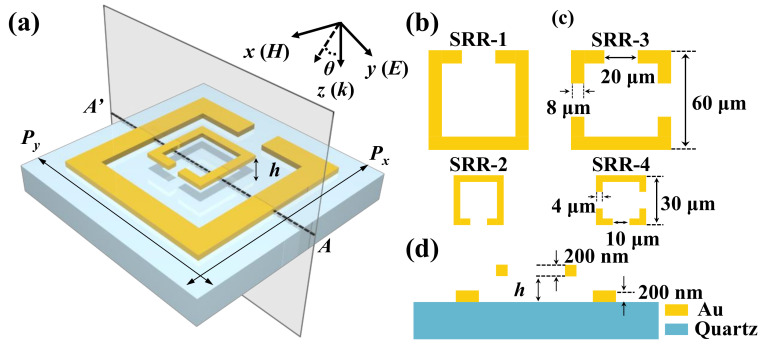
(**a**) Schematic drawing of TTM-1; (**b**) and (**c**) top-views of SRR-1 to SRR-4 and the corresponding geometrical parameters; (**d**) cross-sectional view of TTM-1 along AA’ line in (**a**).

Figure 1a shows the schematic diagram of TTM-1, which is composed of two concentric SRRs. The coordinates of the incident electromagnetic wave are also illustrated, where *E*, *H*, and *k* are the E-field, H-field, and Poynting vector of electromagnetic wave, respectively. The periods of TTM devices are denoted as *P_x_* and *P_y_* along *x*-axis and *y*-axis directions, respectively, which are kept as constant as *P_x_* = *P_y_* =100 μm. The numerical simulation of the proposed device is based on the Lumerical Solution’s finite difference time domain (FDTD) solution. The propagation direction of the incident light is set perpendicular to the *x-y* plane, the polarization angle is set to 0°, and the periodic boundary conditions are adopted along the *x*- and *y*-axis directions, while the *z*-axis direction is the boundary condition of the perfectly matched layer (PML). The resonant frequency monitor is arranged below the device to calculate the transmission spectrum. The permittivities of Au and quartz materials are set as 10^4^ for Au layer and 10 for quartz substrate, respectively. Figure 1b,c show the schematics of TTM-1 composed of SRR-1 and SRR-2, TTM-2 composed of SRR-3 and SRR-4, respectively. The lengths, line widths, and gaps of outer and inner SRRs are defined as constant as 60 μm (outer length), 30 μm (inner length), 8 μm (outer line width), 4 μm (inner line width), 20 μm (outer gap), and 10 μm (inner gap), respectively. Figure 1d shows the cross-sectional view of TTM device along AA’ line of Figure 1a. Two concentric SRRs are composed of tailored gold (Au) layers with 200 nm in thickness on quartz substrate. The distance between SRR-1 and SRR-2 for TTM-1 and between SRR-3 and SRR-4 for TTM-2 is defined as *h*.

## 3. Results and Discussions

**Figure 2:**
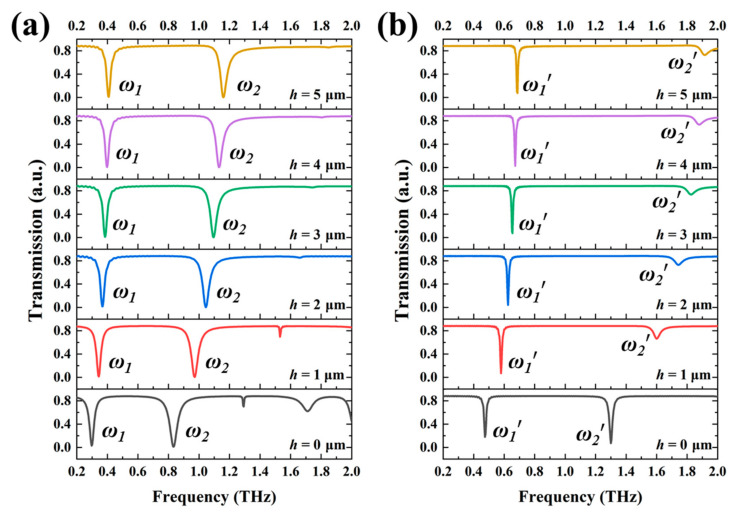
Transmission spectra of (**a**) SRR-1 and (**b**) SRR-2 by changing *h* parameter from 0 μm to 5 μm.

**Figure 3:**
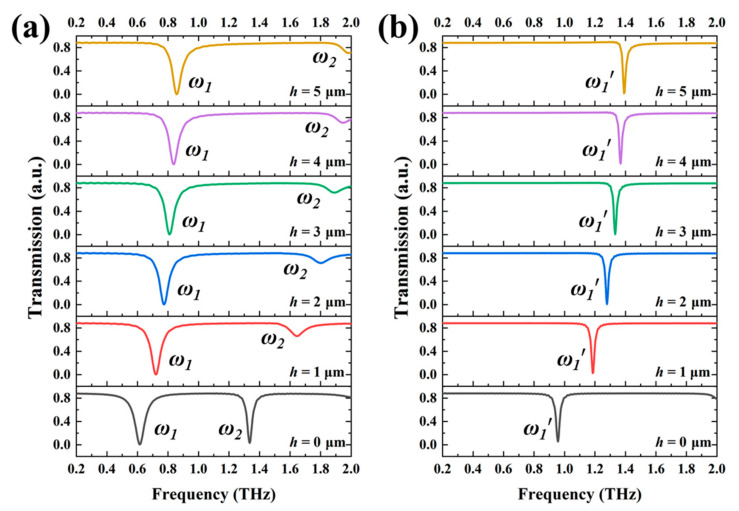
Transmission spectra of (**a**) SRR-3 and (**b**) SRR-4 by changing *h* parameter from 0 μm to 5 μm.

Figure 2 and Figure 3 show the transmission spectra of four kinds of SRRs by changing *h* values from 0 μm to 5 μm. It can be clearly observed that SRRs exhibit dual-resonance characteristic except SRR-4 exhibits single-resonance characteristic in the frequency range of 0.2 THz to 2.0 THz. By changing *h* values from 0 μm to 5 μm, the first resonances of four SRRs are blue-shifted from 0.297 THz to 0.406 THz (SRR-1), 0.474 THz to 0.680 THz (SRR-2), 0.615 THz to 0.857 THz (SRR-3), and 0.958 THz to 1.390 THz (SRR-4), respectively. The tuning ranges are 0.109 THz, 0.213 THz, 0.242 THz, and 0.432 THz for SRR-1, SRR-2, SRR-3, and SRR-4, respectively. While the second resonances of SRR-1, SRR-2, and SRR-3 are blue-shifted from 0.831 THz to 1.160 THz, 1.300 THz to 1.913 THz, and 1.332 THz to 1.986 THz with the tuning ranges of 0.329 THz (SRR-1), 0.613 THz (SRR-2), and 0.654 THz (SRR-3), respectively. However, the second resonant intensities of SRR-2 and SRR-3 are greatly reduced by elevating SRR structures from the substrate. The tuning ranges of second resonances are larger than those of first resonances. In addition, tuning ranges of electric SRRs are larger than those of SRRs under the same feature sizes. It means that electric SRRs are more sensitive to the change of *h* value and more suitable for the design of actively tunable THz device. The physical mechanism of SRR could be explained by the coupling theory. The induced charge will be generated within the gap of SRR by the incident electromagnetic wave shining on the SRR and eventually forming an electric field. Afterward, the movement of charges will generate a ring current and an inductance. Therefore, the resonant frequency of electromagnetic response could be expressed by Drude–Lorentz mode [18]:

(1) $$\omega_{LC}=\frac{1}{\sqrt{LC}}=\left(\frac{c_{0}}{a\sqrt{\varepsilon_{C}}}\right)\sqrt{\frac{g}{w}}$$

where *c_0_* is the velocity of light in vacuum. The gap of SRR is a parallel plate capacitor calculated by *C = ε_0_ε_C_wd/g* and the inductance of SRR is a solenoid expressed as *L* =*μ_0_a^2^/d*, where *w* is the line width, *g* is the gap of split, *d* is the thickness of the metal and *a* is the size of the SRR, *ε_0_* is the free-space permittivity, and *ε_C_* is the relative permittivity of the material in the gap. According to Equation (1), the resonance caused from LC mode can be referred to *L* and *C*, which are related to the geometric parameters and working environment of metamaterial structure. The change of *h* value results in the variation of *ε_C_*, which is in the direct proportion to *Kε_air_* + (1 - *K*)*ε_sub_*, where *ε_air_* is the permittivity of the air, *ε_sub_* is the permittivity of the substrate, and *K* is the filling factor of air. Therefore, by increasing the *h* value, the air is occupied more portion of SRRs. When *K* value is increased and that *ε_sub_* is greater than *ε_air_*, which is caused the *ε_C_* value decreased and then the resonance can be modified and blue-shifted. The transmittance of electromagnetic wave can be expressed by [44]

(2) $$T=\frac{4n_{air}n_{sub}n_{EM}^{2}}{{\left(n_{air}n_{sub}+n_{EM}^{2}\right)}^{2}}$$

where $n_{EM}=\sqrt{\mu \left(\omega \right)\cdot \varepsilon \left(\omega \right)}$, $n_{air}$ and $n_{sub}$ are the refractive index of air and substrate, respectively.

(3) $$\mu \left(\omega \right)=1-\frac{F\cdot \omega_{pm}^{2}}{\omega^{2}-\omega_{LCm}^{2}}$$

(4) $$\varepsilon \left(\omega \right)=1-\frac{F\cdot \omega_{pe}^{2}}{\omega^{2}-\omega_{LCe}^{2}}$$

Drude–Lorentz model is a common method to describe the material properties for the permittivity and permeability [45], which are the resonant frequency of a harmonic oscillator to external frequency-dependent perturbation. $\omega_{p}$ is the plasma frequency, $\omega_{LC}$ is the resonant frequency, and *F* is a dimensionless quantity. These parameters with subscript *e* for electric or *m* for magnetic responses.

**Figure 4:**
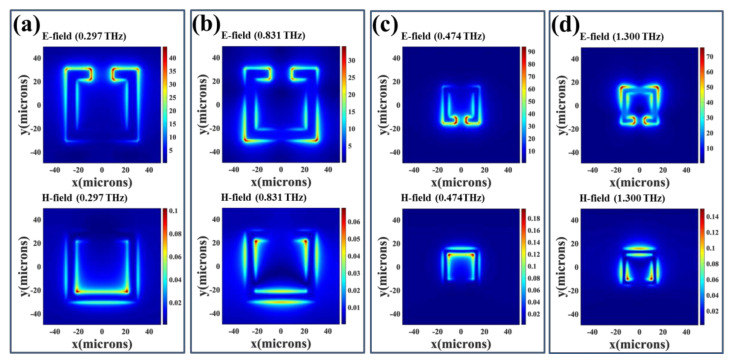
E- and H-field distributions of SRR-1 and SRR-2 with *h* = 0 μm. They are (**a**) SRR-1 monitored at *f* = 0.297 THz; (**b**) SRR-1 monitored at *f* = 0.831 THz; (**c**) SRR-2 monitored at *f* = 0.474 THz and (**d**) SRR-2 monitored at *f* = 1.300 THz; respectively.

**Figure 5:**
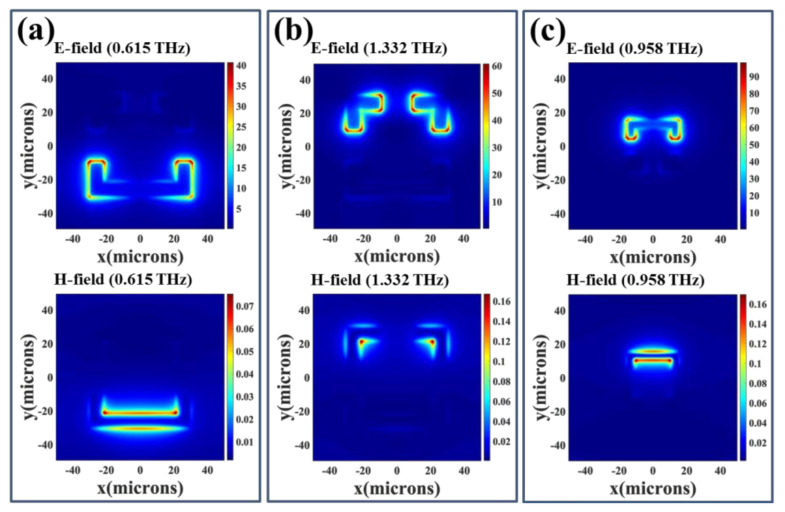
E- and H-field distributions of SRR-3 and SRR-4 with *h* = 0 μm. They are (**a**) SRR-3 monitored at *f* = 0.615 THz; (**b**) SRR-3 monitored at *f* = 1.332 THz and (**c**) SRR-4 monitored at *f* = 0.958 THz; respectively.

To further investigate the physical mechanism of electromagnetic responses of four SRRs, the corresponding E- and H-field distributions of SRRs with *h* = 0 μm are indicated in Figure 4 and Figure 5. There are two resonances for SRR-1, SRR-2, and SRR-3, which are induced by the incident electromagnetic waves. One resonance is LC mode at low frequency band and the other resonance is dipole mode at high frequency band. Figure 4a,c are the E- and H-field energies of LC resonances at 0.297 THz and 0.474 THz for SRR-1 and SRR-2, respectively. The E-field energies are concentrated within the gap. While the H-field energies are mainly distributed in the inside corner opposite the gap. There is a circulating current on the metal ring surface and its effect can be equivalent to a LC circuit model to analyze first resonance. This resonance is called LC resonance. Figure 4b,d are the E- and H-field energies of dipole resonances at 0.831 THz and 1.300 THz for SRR-1 and SRR-2, respectively. The E-field energies are mainly distributed within the gap and outside of SRR corner, while the H-field energies are mainly distributed in the inside of SRR corner. There are three different circulating currents on the surface of the metal ring to form an electric dipole resonance. In the same way, the physical mechanism of resonance between electric SRR and SRR is similar. SRR-3 has a LC resonance and a dipole resonance, while SRR-4 only has a LC resonance in the frequency range of 0.2 THz to 2.0 THz. The E- and H-field distributions of LC resonances at 0.615 THz for SRR-3 and 0.958 THz for SRR-4 are shown in Figure 5a,c. The E- and H-field energies are mainly distributed along the U-shape structure. The field distributions of dipole resonance of SRR-3 at 1.332 THz are shown in Figure 5b. The E- and H-field energies are mainly distributed in the gap areas and inside of corners, respectively. 

**Figure 6:**
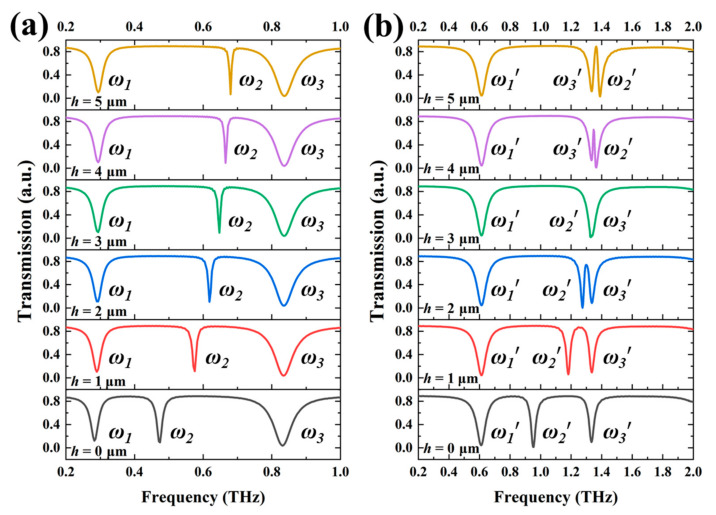
Transmission spectra of (**a**) TTM-1 and (**b**) TTM-2 with different *h* values.

Since the mechanism of SRRs with different *h* values shows the tunability, they can be further designed to perform the tunable TTM devices by combining SRR-1 and SRR-2 together to form TTM-1, while combining SRR-3 and SRR-4 together to form TTM-2. The resonances of TTM-1 are superimposed by those of SRR-1 and SRR-2, while the resonances of TTM-2 are superimposed by those of SRR-3 and SRR-4. Figure 6 shows the transmission spectra of TTM-1 and TTM-2 by changing *h* values of SRR-2 and SRR-4 from 0 μm to 5 μm. There are three resonances under the condition of *h* = 0 μm, the first resonance (0.283 THz for TTM-1, 0.615 THz for TTM-2) and third resonance (0.831 THz for TTM-1, 1.332 THz for TTM-2) are almost kept as stable. The second resonances are blue-shifted from 0.474 THz to 0.680 THz for TTM-1 and 0.958 THz to 1.390 THz for TTM-2, respectively. TTM-1 can tune the second resonance with a tuning range of 0.206 THz by changing the *h* value from 0 μm to 5 μm and the first and third resonances are stable. TTM-2 can tune the second resonance with a tuning range of 0.432 THz by changing *h* value from 0 μm to 5 μm and the first and third resonances are stable. It is worthy to mention that the second resonance of TTM-2 will be merged with third resonance when *h* = 0 μm, then exhibit electromagnetically induced transparency (EIT) characteristic when the *h* value becomes larger than *h* = 3 μm. Owing to the tunable second resonance of TTM-2, the EIT resonance of TTM-2 can be switched on and off states by changing *h* value from 3 μm to 4 μm and then that can be tuned from 1.347 THz to 1.365 THz by changing *h* value from 4 μm to 5 μm. For the stable first and third resonances of TTM-1 and TTM-2, they are suitable to be used as an optical reference signal to monitor and improve the sensitivity and efficiency of optical sensors. The resonance shifts of the second resonances of TTM-1 and TTM-2 are similar to those of the LC resonances of SRR-2 and SRR-4, which mean that there are no coupling effects between two SRR structures. The method proposed in this study can select one of the resonances shifting, and the other resonances remain stationary, which can be realized by controlling the *h* value. We take TTM-1 as an example, which can realize the tuning of single-, dual-, or triple-resonance, respectively. When the inner SRR is elevated, there is only one resonance shifting in the range of 0 to 1 THz, while the other two resonances are stationary. When the outer SRR is elevated, there are two resonances shifting, and one resonance is stationary. When the whole SRRs are elevated, three resonances will be shifted simultaneously. In addition, the proposed device can be tuned continuously and linearly by changing *h* value, and it does not require extra equipment to stimulate, which is convenient for miniaturization.

**Figure 7:**
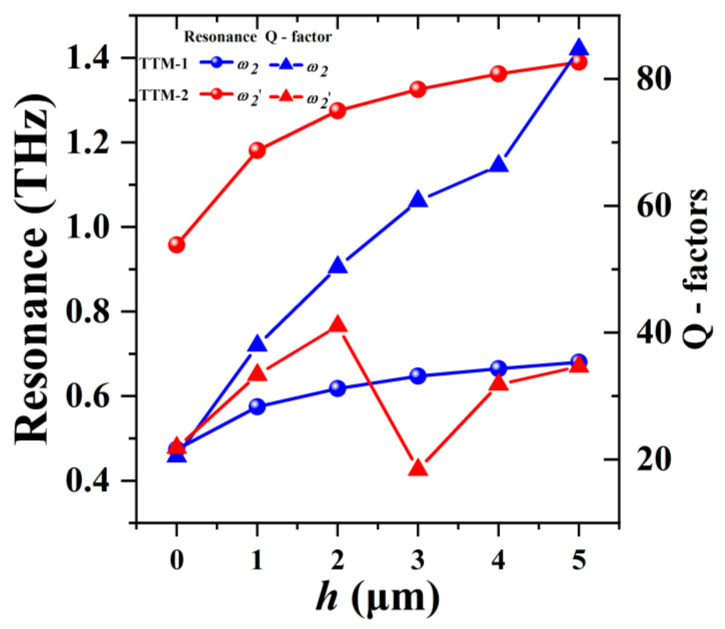
Relationships of resonances and Q-factors with different *h* values of TTM-1 and TTM-2.

The relationships of resonances and *h* values of TTM-1 and TTM-2 are summarized in Figure 7. By changing the *h* value from 0 μm to 1 μm, the blue shifts of *ω_2_* and *ω_2_*' are 0.101 THz and 0.223 THz respectively. With the increased *h* value, the frequency shift is gradually decreasing. Thus, the frequency shift of *ω_2_* and *ω_2_'* tend to saturate after 3 μm. Q-factor of resonance is used to evaluate the key parameter of the sensing performance, which is defined as Q = *f*/FWHM, where *f* is the resonance and FWHM is the full width of half maximum of resonance. The higher Q-factor represents the sharper resonance, which indicates that the loss of the resonance system is minor. In Figure 7, the Q-factors of TTM are increased by increasing *h* value. When the *h* value is changed from 0 μm to 5 μm, the corresponding Q-factors of *ω_2_* of TTM-1 are 20.5, 37.96, 50.35, 60.77, 66.31, and 84.70, respectively. For *ω_2_*′ of TTM-2, Q-factors are calculated as 21.88, 33.34, and 41.07 for the conditions of *h* = 0, 1, and 2 μm. Afterwards, it decays to 18.40 when *h* = 3 μm and finally increases to 31.83 and 34.66 for *h* = 4 μm and 5 μm, respectively. The maximum Q-factor is 84.70 at *ω_2_* under the condition of *h* = 5 μm for TTM-1, which is very suitable for the use in sensing application with high-sensitivity. In order to assess how the TTM device is effective, the dephasing time of induced resonances is significant, the dephasing time is given by *T_d_* = 2ℏ/FWHM, where ℏ is the reduced Planck constant [46]. The average dephasing time of *ω_2_* and *ω_2'_* are calculated as 1.14 × 10^−11^ s and 3.23 × 10^−12^ s.

**Figure 8:**
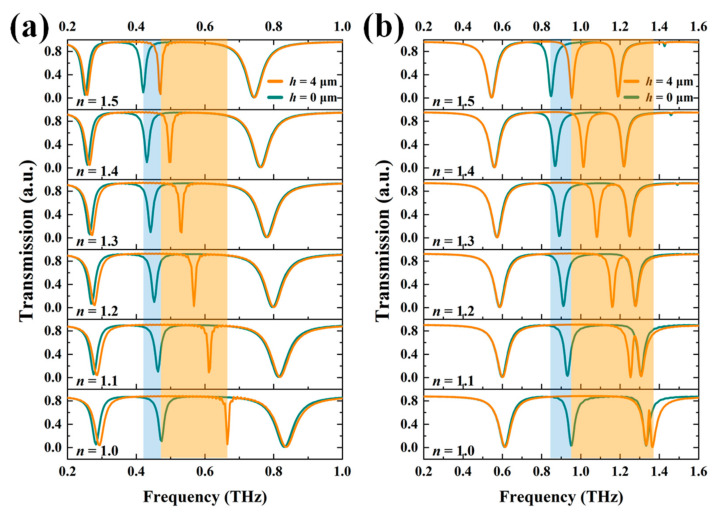
Transmission spectra of (**a**) TTM-1 and (**b**) TTM-2 with different *n* values when *h* = 0 μm and *h* = 4 μm. The blue and orange area are the resonant shifts when *h* = 0 μm and *h* = 4 μm, respectively.

**Figure 9:**
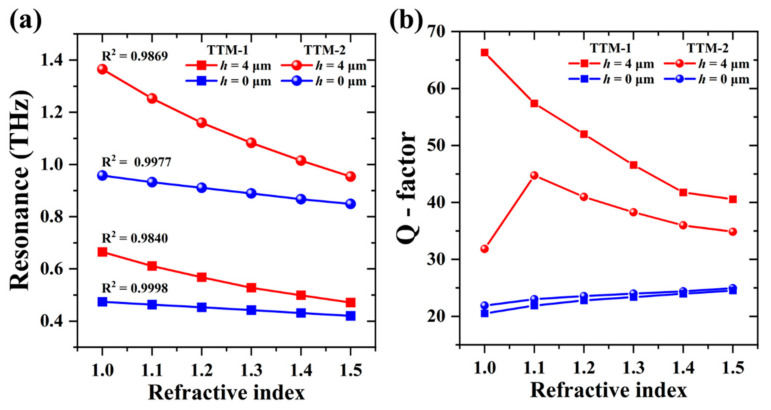
Relationships of (**a**) resonances and (**b**) Q-factors with different *n* values of TTM-1 and TTM-2.

To further investigate the practical applications of TTM devices, TTM-1 and TTM-2 are exposed on the surrounding ambient with different refraction index (*n*). Figure 8a,b show the transmission spectra of TTM-1 and TTM-2 with *h* = 4 μm (orange line) and *h* = 0 μm (blue line) exposed on different *n* values, respectively. The second resonance is generated from the inner SRR and red-shifted by increasing *n* value from 1.0 to 1.5. The first and third resonances are shifted slightly. The resonance shifts of TTM-1 and TTM-2 with *h* = 4 μm are larger than those with *h* = 0 μm as the orange and blue areas shown in Figure 8. It means that TTM devices are more sensitive to the change of ambient *n* value at the condition of *h* = 4 μm. In Figure 8b, the EIT resonance generated by the resonance of inner SRR shifts from 1.365 THz to 1.253 THz when *n* changes from 1.0 to 1.1. The relationships of resonances and *n* values are summarized in Figure 9a. The linearity is defined as the value of coefficient of determination (R^2^), which can evaluate the fitting degree of regression equation. The calculated values of R^2^ are 0.9869, 0.9977, 0.9840, and 0.9998 for four curves from top to bottom as shown in Figure 9a. These higher fitting degrees between regression lines and simulated values indicate the high linearity of the relationship, which means the proposed TTM devices are quite suitable for sensing applications. Figure 9b shows the summaries of Q-factors and different *n* values of TTM-1 and TTM-2. The sensing capability of the proposed TTM devices is evaluated by using the definitions of sensitivity (*S*) and figure of merit (FOM), which are defined as [47]

(5) $$S=\frac{\Delta f}{\Delta n}\;(\mathrm{THz}/\mathrm{RIU})$$

(6) $$\mathrm{FOM}=\frac{S}{\mathrm{FWHM}}$$

where *f* is the resonance, *n* is the refractive index, and FWHM is full width of half-maximum. *S* value represents the change of resonance per unit *n* value, and FOM value considers the factor of bandwidth. The higher FOM value indicates the better sensing performance of the device. The sensing capabilities of TTM-1 and TTM-2 are summarized in Table 1. The sensing performance of the proposed TTM devices with *h* = 4 μm is much better than that with *h* = 0 μm. The maximum values of *S* and FOM are 0.54 THz/RIU and 50.69 for TTM-1 while those are 1.12 THz/RIU and 39.98 for TTM-2. Herein, TTM-1 has smaller *S* value but higher FOM value as its Q-factor is higher than that of TTM-2 when *h* = 4 μm. These values are better than those of metamaterial-based sensors reported in the literature papers [48,49,50,51] as summarized in Table 2. Q-factors, *S*, and FOM values of the proposed TTM devices are greatly improved by elevating the inner SRRs. It means that the sensing performance of the THz metamaterial-based device can be manually improved by properly arranging the geometric position of meta-atoms. Additionally, such designs can be applied in many fields according to the real requirement, such as tunable filters, frequency-selective detectors, and tunable high-efficient sensors.

**Figure 10:**
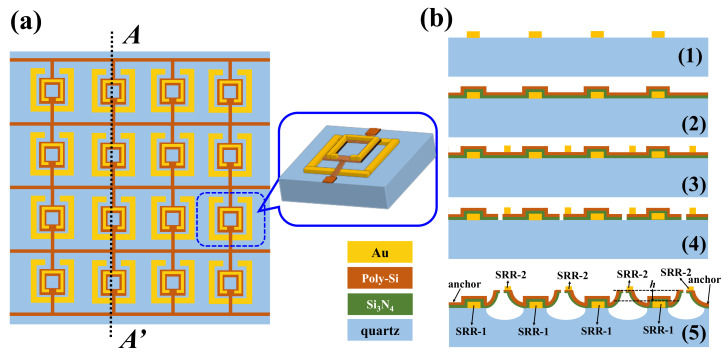
Schematic drawing of (**a**) proposed MEMS-based TTM-1 array; (**b**) Fabrication process of proposed TTM-1 using MEMS technique along AA’ line in (**a**); (**b1**) The SRR-1 structures are deposited and patterned by using lift-off and sputtering processes sequentially; (**b2**) The poly-Si and Si_3_N_4_ thin-films are deposited on sample surface using PECVD process sequentially. (**b3**) The SRR-2 structures are deposited and patterned by using lift-off and sputtering processes sequentially. (**b4**) Sample is patterned by using photolithography and RIE processes to define the ETAs structures. (**b5**) Sample is released the ETAs structures using VHF to perform the vertical tuning mechanism of TTM-1.

In this study, the proposed MEMS-based TTM devices exhibit tunable resonance characteristic which could be realized by using MEMS technique. We take TTM-1 as an example to describe the fabrication process and its mechanism. Figure 10a shows the schematic drawing of proposed TTM-1 array. The elevated SRR-2 structures are supported by using a membrane composed of poly-Si and Si_3_N_4_ bilayer. This bilayer is no influence for the resonance of TTMs in the THz frequency range owing to it is transparent for THz wave [52]. The elevated structures are supported by poly-Si/Si_3_N_4_ bilayer using residual stress induced electrothermal actuators (ETAs). The thermal expansion coefficients of poly-Si and Si_3_N_4_ layers are mismatched. As the structures are released, the poly-Si layer is under tensile residual stress due to its larger thermal expansion coefficients while the Si_3_N_4_ layer is under compressive residual stress due to its smaller thermal expansion coefficients. Such initial stress between poly-Si and Si_3_N_4_ layers makes the cantilever deformed out-of-plane and bending upward. By applying a DC bias voltage on these residual stress induced ETAs, the electrothermal attraction force is induced between the released cantilevers. Meanwhile, the out-of-plane bilayer cantilever will be bended downward to the substrate owing to such electrothermal attraction force. Therefore, TTM-1 can be tuned vertically by actuating the ETAs to change *h* value. The corresponding fabrication process of TTM-1 is illustrated in Figure 10b. First, the SRR-1 structures are deposited and patterned by using lift-off and sputtering processes sequentially as shown in Figure 10(b1). Second, the poly-Si and Si_3_N_4_ thin-films are deposited on sample surface using plasma enhanced chemical vapor deposition (PECVD) process sequentially as shown in Figure 10(b2). Third, the SRR-2 structures are deposited and patterned by using lift-off and sputtering processes sequentially as shown in Figure 10(b3). Fourth, sample is patterned by using photolithography and reactive ion etching (RIE) processes to define the ETAs structures (Figure 10(b4)). Finally, sample is released the ETAs structures using vapor HF (VHF) to perform the vertical tuning mechanism of TTM-1 as shown in Figure 10(b5). 

## 4. Conclusions

In conclusion, we present two designs of TTM based on concentric SRRs to be used as refractive index sensors with high sensitivity and FOM. By elevating the inner SRR, the resonance of TTM-1 can be tuned from 0.474 THz to 0.680 THz while that of TTM-2 can be tuned from 0.958 THz to 1.390 THz. In addition, the multiple resonances can be tuned by elevating the outer SRR or the entire SRR structures. It indicates that TTM can be potentially used as tunable filter by using MEMS technique to modify the electromagnetic response of TTM with reconfigurable geometrical dimensions. By increasing the *h* value, the sensing performance of TTM can be improved. The maximum sensitivity and FOM values of TTM-1 are 0.54 THz/RIU and 50.69, respectively. While the maximum sensitivity and FOM values of TTM-2 are 1.12 THz/RIU and 39.98, respectively. These unique optical properties of TTM provide the potential opportunity for widespread applications in filters, modulators, gas sensors, biosensors, and environmental sensors.

## Figures and Tables

**Table 1:** Sensing capabilities of TTM-1 and TTM-2 with different ambient n values.

*n*	*f* (THz)	FWHM (THz)	Q-factor	*S* (THz/RIU)	FOM
TTM-1 (*h* = 0 μm/4 μm)					
**1.0**	0.474/0.665	0.023/0.010	20.50/66.31	—	—
**1.1**	0.463/0.611	0.021/0.011	21.89/57.38	0.11/0.54	5.20/50.69
**1.2**	0.453/0.568	0.020/0.011	22.78/51.98	0.11/0.43	5.54/39.36
**1.3**	0.441/0.532	0.019/0.011	23.38/46.57	0.11/0.36	5.82/31.52
**1.4**	0.431/0.496	0.018/0.012	23.96/41.76	0.11/0.36	6.12/30.32
**1.5**	0.420/0.471	0.017/0.012	24.52/40.65	0.11/0.25	6.42/21.60
**TTM-2 (*h* = 0 μm/4 μm)**					
**1.0**	0.950/1.365	0.043/0.043	21.88/31.83	—	—
**1.1**	0.932/1.253	0.041/0.028	23.01/44.73	0.18/1.12	4.44/39.98
**1.2**	0.911/1.160	0.038/0.028	23.55/40.97	0.22/0.94	5.69/33.21
**1.3**	0.889/1.080	0.037/0.028	23.99/38.27	0.22/0.79	5.94/27.99
**1.4**	0.867/1.015	0.036/0.028	24.38/35.97	0.22/0.65	6.18/23.03
**1.5**	0.849/0.954	0.034/0.027	24.92/34.84	0.18/0.61	5.28/22.28

**Table 2:** Comparison of the proposed sensor with prior presented sensors.

Design	Q-factor	*S* [THz/RIU]	FOM
Ref. [48]	30.5	0.280	8.54
Ref. [49]	22.1	0.300	2.94
Ref. [50]	38.9	0.104	3.00
Ref. [51]	58.0	0.105	7.50
TTM-1	57.4	0.540	50.7
TTM-2	44.7	1.120	40.0

## Data Availability

No new data were created or analyzed in this study. Data sharing is not applicable to this article.
